# Spatial distribution of short birth interval and associated factors among reproductive age women in Ethiopia: spatial and multilevel analysis of 2019 Ethiopian mini demographic and health survey

**DOI:** 10.1186/s12884-023-05610-9

**Published:** 2023-04-22

**Authors:** Sisay Yitayih Kassie, Habtamu Setegn Ngusie, Addisalem Workie Demsash, Tilahun Dessie Alene

**Affiliations:** 1 Department of Health informatics, College of Health Sciences, Mettu University, P.O.Box:318, Mettu, Ethiopia; 2 Department of Health informatics, School of Public Health, College of Medicine and Health Sciences, Woldia University, Woldia, Ethiopia; 3 Department of Pediatrics and Child Health, School of Medicine, College of Medicine and Health Sciences, Wollo University, Dessie, Ethiopia

**Keywords:** Short, Birth, Interval, Reproductive age, Women, Spatial variation, Ethiopia

## Abstract

**Background:**

Short Birth Interval negatively affects the health of both mothers and children in developing countries. Studies conducted in Ethiopia on the spatial variation and determinants of individual and community-level factors about short birth intervals were limited. Thus, this study was intended to assess the spatial variation of the short birth interval and its determinants in Ethiopia.

**Methods:**

This study is a secondary analysis of the Ethiopian Demographic and Health Survey (mini EDHS 2019). A total of 1784 reproductive-age women were included in the analysis. The global spatial autocorrelation (Global Moran’s I) and the Getis-Ord statistics tool were used to detect the presence of clustering and the high/low hotspot areas of SBI respectively. Ordinary kriging was used to interpolate short birth intervals, and spatial scan statistics were employed to identify spatial clusters with high and low SBI. A multilevel multivariable model was used to identify predictors of a short birth interval.

**Results:**

The prevalence of SBI was 62.89% (95%CI: 59.3, 69.7) in Ethiopia. High clustering of SBI was observed in all parts of Somali, in Afar (zones 1, 3, 4, &5), Oromia (Guje, Bale, & West Harerge), and northern Tigray. The most likely significant primary cluster was observed in the Somali region. Women who lived in the primary cluster were 24% more likely to have a short birth interval than those who lived outside the window. In the multilevel mixed-effect analysis age 25–34 [(AOR = 0.40, 95% CI: 0.35, 0.45)], 35–49 [(AOR = 0.44, 95% CI: 0.38, 0.51)], Muslim religion follower [(AOR = 3.5, 95% CI: 2.7, 4.69)], no formal education [(AOR = 0.5, 95% CI: 0.37, 0.70)], primary education[(AOR = 0.4, 95%CI: 0.28, 0.53)], and secondary education [(AOR = 0.3, 95% CI: 0.24, 0.48)], middle [(AOR: 1.3, 95% CI: 1.2, 1.52)], rich wealth status [(AOR: 1.4, 95% CI: 1.3, 1.68)], female sex children [(AOR: 1.2, 95% CI: 1.09, 1.42)], and two or fewer ideal number of children [(AOR = 0.2, 95% CI: 0.25, 0.32)] were found to be significant predictors of SBI.

**Conclusion:**

Overall, SBI was high and significantly clustered across the region of Ethiopia. Age, religion, education, wealth status, the sex of the indexed child, and the ideal number of children were found to be significantly associated with short birth intervals. Hence, the government should design a health promotion strategy and public health awareness in the identified hotspot areas of SBI and should scale up family planning and the wealth status of reproductive-age women.

## Background

According to the World Health Organization (WHO), a short birth interval (SBI) is defined as the time of less or less than 24 months between births and the next pregnancy [1], or a period of fewer than 33 months between two consecutive live births [2], which have a negative outcome on maternal, perinatal, and neonatal outcomes, as well as on child health [3].

Short birth interval has become a public health and demographic concern and is associated with several adverse health outcomes, such as: poor maternal nutritional status and folate depletion, suboptimal lactation for the newborn, cervical insufficiency, infections, sibling competition, incomplete healing of the uterus, and abnormal remodelling of endometrial blood vessels [1, 4]. It is also associated with a higher risk of maternal morbidities [5, 6], low birth weight [7], preterm birth [8, 9], congenital anomalies [10], autism [11], high risk of blood pressure, maternal morbidities, and infant mortality [1, 12, 13]. Nearly 33% of short birth intervals occur in Central Asian States, and it is 57% in Sub-Saharan Africa [14] and 53% in Ethiopia. A short birth interval increases infant mortality by 60% and is the major cause of around 822 maternal deaths a day in low- and middle-income countries [15, 16]. Recent research evidence suggests improving birth intervals for three or more years helps to reduce maternal mortality, infant mortality by 50%, and child health [1, 2, 17, 18]. One estimate suggests that around 2 million of the 11 million deaths per year of children under 5 could be prevented by avoiding birth intervals [19]. As a result, international bodies such as the WHO and United States Agency for International Development (USAID) have called for further research and actions to address short birth intervals [20, 21]. However, developing countries experienced a high number of neonatal and infant mortality, especially in Ethiopia, and birth interval practice vary widely around the world [18, 22].

Studies done using the Demographic and Health Survey indicate the high prevalence of SBI in African countries such as Uganda (25.3%), Rwanda (20%), and Cameron (21.3%) [23]. In Ethiopia, according to the EDHS trend, the prevalence of short birth intervals among regions of Ethiopia ranges from 0.2% in Gambella and Harari Region to 50.9% in Oromia Region [24]. Another previous study in Ethiopia showed the prevalence of SBI was 43.4% [25], 45.7% [26], 46% [27], 23.3% [28], and 57% [29]. Practical evidence has documented young maternal age [1, 30], maternal education [30–33], household wealth status [34, 35], rural place of residence [32, 35], having fewer children [33], the preceding child being female [33, 34, 36–38], the death of the preceding child [37, 39], Sub-optimal breastfeeding [40, 41], no use of modern contraceptives [34, 35, 37], the husband’s education, sociocultural factors, and religion [33, 34, 42–44] were identified as associated factors. Despite the increasing trend in contraceptive utilization in Ethiopia, from 5.9% in 2000 to 14.0% in 2005, 27.0% in 2011, and 35.0% in 2016, the prevalence of short birth interval has remained unchanged [45, 46].

When we comes to Ethiopia, most of the previous studies conducted in Ethiopia did not examine the extent of the spatial variation within and between regions, zones (districts), and predictors of short birth intervals to make specific area-based evidence-based decisions. The previous studies done in Ethiopia using DHS data were focused purposefully on selected areas expected to have high SBI. This lacks the generalizability of the result across the country to design and implement an evidence-based medicine public health intervention. Besides, the previous studies used a less advanced model to identify factors that influence SBI. This lacks the ability to identify which level of factors affect SBI and further limits policymakers and program managers areas of focus to reduce the risks.

Hence, a spatial multilevel analysis will help to understand significant high-risk areas and predictors of short birth intervals and will also help to evaluate the quality of maternal health service. Therefore, this study aimed to assess the spatial distribution and predictors of short birth intervals in Ethiopia. The findings of this study will help policymakers, program planners, and other stakeholders in guiding health investment and developing prevention and intervention programs. In addition, mapping or exploring short birth interval hot spots will provide a deeper understanding of the impact of past intervention strategies, including family planning services in each region.

## Methods and materials

### Study design and setting

This study used data from the Ethiopian Demographic and Health Survey (DHS), collected using across-sectional study design. Ethiopia, the second most populated nation in Africa with 114.96 million population. Ethiopia is located in the Horn of Africa between latitudes 3 and 15 degrees north and 33 and 48 degrees east. Nine regional states (Tigray, Afar, Amhara, Oromia, Somali, Benishangul-Gumuz, Southern Nations Nationalities and People (SNNP), Gambella, and Harari) and two city administrations make up its administrative organization (Addis Ababa and Dire Dawa). These are subdivided into administrative Woredas and further divided into Kebele, which is the smallest administrative unit in the country.

### Data source, extraction, sampling procedure, and study participants

Our data source was the mini-EDHS survey, which was collected in 2019. EDHS is collected every 5 years by the Ethiopian Central Statistical Agency (CSA) along with ICF International and funded by USAID. The data sets for EDHS were downloaded in Stata format with permission from the Measure DHS website (http://www.dhsprogram.com).

The shape file of the map of Ethiopia has been accessed as an open source without restriction from the Open Africa website (https://africaopendata.org/dataset/ethiopia-shapefiles). The survey was conducted from March 21, 2019, to June 28, 2019. The census frame is a complete list of the 149,093 EAs created for the 2019 EPHC. An EA is a geographic area covering an average of 131 households. It employed a two-stage stratified selected sample. In the first stage, a total of 305 EAs (93 in urban areas and 212 in rural areas) were selected with probability proportional to EA size (based on the 2019 EPHC frame) and with independent selection in each sampling stratum.

**Fig. 1:**
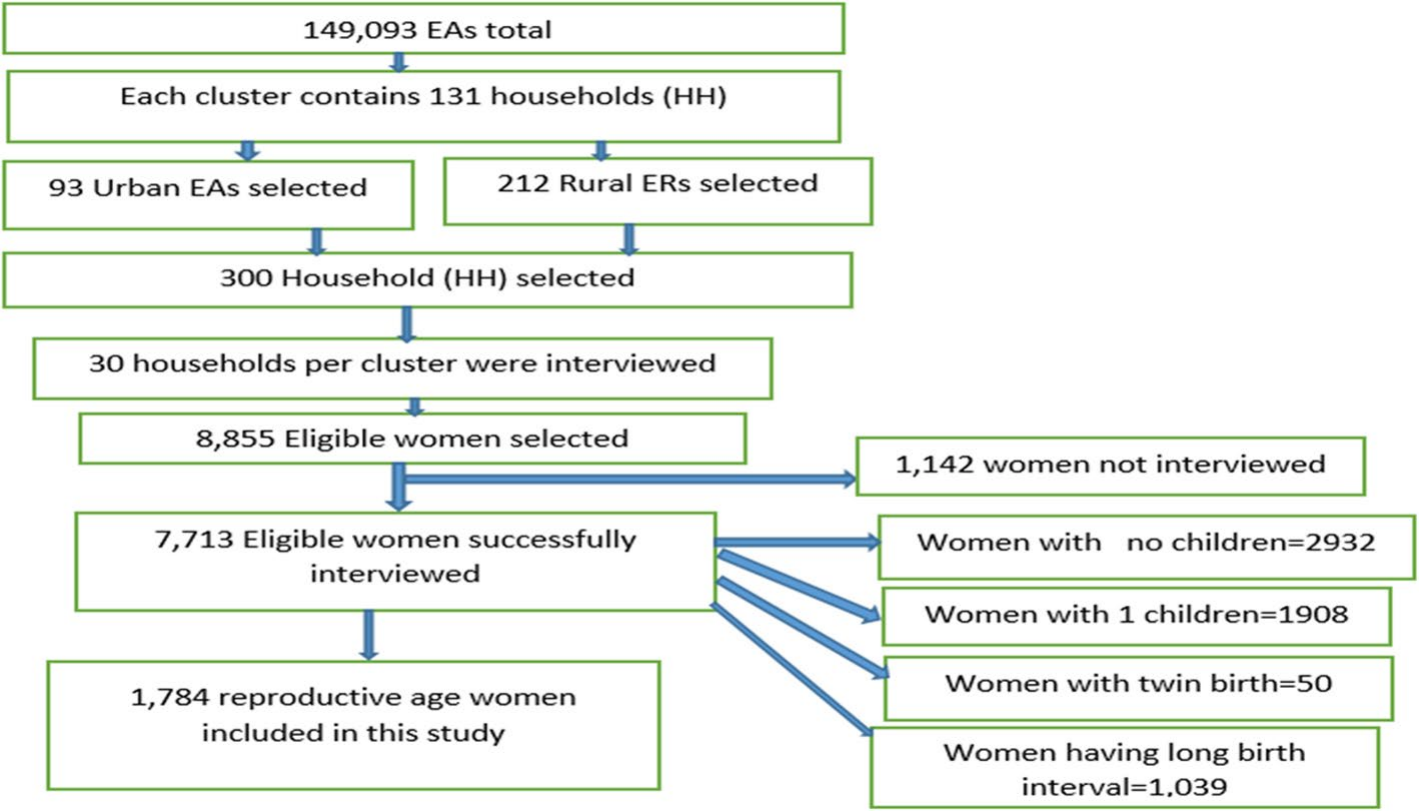
Sampling procedure extracted for reproductive age women from Ethiopian Demographic and Health Survey 2019

In the second stage of selection, a fixed number of 30 households per cluster were selected with an equal probability of systematic selection from the newly created household listing. All women aged 15–49 who were either permanent residents of the selected households or visitors who slept in the household the night before the survey were eligible to be interviewed. The detailed sampling procedures were presented in the 2019 EMDHS report from the DHS website (https://www.dhsprogram.com). In this study, all reproductive aged women 15–49 were a source population. Women who had at least two consecutive live births in the 5 years preceding the survey were the study population. All women of childbearing age [13–19, 21–25, 27–49] having multiple birth intervals since the preceding pregnancy that ended in a live birth (birth interval > 5 years), having no children, having a first birth order, and having twins were excluded. Ultimately, a total representative sample of 1784 reproductive-age women were included in the respective survey. Geographic coordinates of each survey cluster were also collected using Global Positioning System (GPS) receivers. To ensure confidentiality, GPS latitude and longitude positions for all surveys were randomly displaced before public release. The detailed procedure has been presented in each EDHS report [17–19, 37] (Fig. 1).

## Variables of study

### Dependent variable

In this study, the dependent variable was the short birth interval. A birth interval of less than 33 months was considered short, while a birth interval of 33–60 months was considered normal. Therefore, it was dichotomized as follows:

$$Yij=\left\{\begin{array}{c}1,\ i\textrm{f}\ \textrm{there}\ \textrm{is}\ \textrm{short}\ \textrm{birth}\ \textrm{interval}\ \\ {}0, if\ there\ is\ normal\ birth\ interval\end{array}\right.$$

When Y is the outcome variable (short birth intervals), i is for the individual level factors, and j is for the community level factors.

### Independent variables

The independent variables were classified into individual-level variables and community-level variables. Individual-level variables were the sex of the index child, age at marriage, mother’s age at first birth, parity (number of live births), women’s education level, husband’s education level, wealth index, respondents’ occupation, religion, survival status of the index child, the ideal number of children, preferred waiting time to birth, number of living children, duration of breastfeeding (in months), and contraceptive utilization. The community-level variables included in this study were region and residence. Region and residence were directly obtained from EDHS.

## Data management and analysis

Data cleaning, labelling, processing, and coding were done using the SATA 15 version. The EDHS dataset was weighted using sampling weights, a primary sampling unit, and strata before any statistical analysis to restore the representativeness of the survey and to take into account the sampling design and get reliable statistical estimates. The descriptive analysis was performed and presented using tables.

### Spatial analysis

ArcGIS 10.8 and SaTScan 9.6 statistical software were employed to explore spatial distribution and determine the geographical locations of statistically significant spatial windows for short birth intervals.

### Spatial autocorrelation and hotspot analysis (Getis-Ord G statistics)

Spatial autocorrelation (Global Moran’s I) is used for detecting the geographical distribution of short birth intervals. The spatial autocorrelation coefficient is statistically significant when tested against the null hypothesis. Moran’s I value ranges from − 1 to 1. The Moran’s I value is close to − 1 indicating “dispersed short birth intervals”, 1 indicating “clustering of short birth intervals”, and zero indicating a “perfect random pattern of short birth intervals”, respectively [47]. If Moran’s I score is zero, then there is no spatial or perfectly random autocorrelation. A statistically significant Moran I-value (*p* < 0.05) results in the rejection of the null hypothesis (short birth intervals were randomized) and indicates the presence of spatial autocorrelation [48]. The Getis-Ord G index was used to determine the spatial heterogeneity (localization and variation) of significant areas of low or high prevalence of short birth intervals for each cluster. It helps to classify autocorrelation into negative or positive autocorrelation. The Z-score and *p*-value statistics were calculated to identify the significant clustering. A Z-score near zero indicates perfect randomness within the study area. A positive Z-score with a *P*-value of > 0.05 indicates statistical clustering of the hotspots of short birth intervals. Thus, the null hypothesis cannot be rejected. Whereas a negative Z-score with a p-value of < 0.05 indicates statistical clustering of reproductive age women who have no short birth intervals.

### Spatial interpolation

The spatial interpolation is used to predict the un-sampled measurement areas in Ethiopia from the observed spatial pattern of short birth intervals in reproductive-age women. The current short birth interval data is used as an input to predict un-sampled areas of the country. Among the different interpolation techniques are: The ordinary Kriging spatial interpolation method was used in this study setting to predict un-sampled areas of short birth interval since they optimized the weight and had a small mean square error and residual [49]. Based on the input data at each location, the semi-variogram model was constructed and used to define the weight, which paternally determines the prediction of new values in areas not sampled. As a result, a new simulated semi-variogram model was generated.

### SaTScan analysis

The Bernoulli-based model was employed to identify statistically significant spatial clusters of short birth intervals using Kuldorff’s SaTScan software version 9.6 [50]. A circular window that moves systematically throughout the study area was used to identify a significant clustering of short birth intervals. For this study, reproductive-age women who experienced short birth intervals were considered a case, and those who did not experience short birth intervals (normal birth intervals) considered as a control to best fit the model for the scanning window. The default maximum spatial cluster size of < 50% of the population was used as an upper limit since it allowed both small and large clusters to be detected and ignored clusters that contained more than the maximum limit. Choosing a cluster size of 50% of the total population is the default option for the maximum size of the scan window. It is often used to search for clusters that are most likely to have a higher likelihood value. Kuldorff’s indicated that a window sized up to 50% of the population at risk can reduce negative clusters (highly sensitive), avoid missing clusters, and be more likely to contain the true significant clusters than the small scanning window.

A Likelihood Ratio (LR) test statistic and a *p*-value were used to determine if the number of observed short birth interval cases within the potential cluster was significantly higher than expected for each potential clusters. The cluster that performed best according to the scanning window’s maximum likelihood was chosen. Each cluster’s p-value was assigned using Monte Carlo hypothesis testing by comparing the rank of the maximum likelihood derived from real data with the maximum likelihood from the random datasets. The primary and secondary clusters were identified and assigned *p*-values. Finally, the clusters were ranked based on their likelihood ratio test, based on 999 Monte Carlo replications [51].

### Summary

The spatial autocorrelation (Global Moran’s I) is used to measure the overall spatial autocorrelation of short birth intervals. It used to indicate “dispersed”, “Clustered,” and “random pattern” of short birth intervals. The Getis-Ord G index were used to identify the spatial heterogeneity (location and variation) of significant prevalence areas of high or low short birth intervals for each cluster.

The spatial interpolation was used to predict the un-sampled measurement areas in Ethiopia from the observed spatial pattern of short birth intervals of reproductive-age women. Furthermore, spatial SaTScan statistical analysis and Bernoulli-based model were employed to identify statistically significant spatial clusters of short birth intervals.

### Multilevel binary logistic analysis

Since the DHS data are hierarchical in nature, women are nested within a cluster, and we expect that women within the same cluster may be more similar to each other than women in another cluster. To account for this cluster effect, we consider a variability test across a cluster since this violates the assumption of the traditional regression model. Due to this, a multilevel logistic analysis was used to determine the effect of the predictors of short birth intervals. Four models, such as: Model 0 or (the empty/null model) was fitted without explanatory variables to test the random variability of short birth intervals in the intercept and to estimate the intra-class correlation coefficient (ICC) and proportional change in variance (PCV), Model 1 (containing individual factors only), Model 2 (community-level factors), and Model 3 (both individual and community-level factors). Akaike Information Criteria (AIC) and Bayesian Information Criteria (BIC) were used in the design and comparison of models. Each successive AIC and BIC value was compared, and the model with the lowest AIC and BIC value was considered the most suitable model. Variables from the first bivariate logistic regression analysis with *p*-values < 0.2 were included in the multilevel analysis. In the final model, variables with p-values< 0.05 were declared to be factors associated with the outcome variable, and the result was presented using an adjusted odd ratio (AOR) with 95% CI.

## Ethical consideration

The data were obtained by requesting it from the Demographic and Health Surveys (DHS) Program and can be freely accessed from the program website (https://www.dhsprogram.com). For this particular study, a brief description of the protocol was submitted to the MEASURE DHS program to access and analyze the data. As a result, we obtained permission to access the EMDHS 2019 data through (https://www.dhsprogram.com/Date/terms-of-use.cfm) for statistical analysis and reporting. During EDHS data collection, informed consent was obtained from each participant, all identifiers were removed, and the confidentiality of the information was maintained.

## Result

### Sociodemographic and other health-related characteristics

From the EMDHS-2019 dataset, a total of 1784 weighted samples of reproductive-age women [13–19, 21–25, 27–49] were included in the study. Out of the total respondents, 1430 (80.16%) women were living in rural areas; 1039 (58.24%) of the women were Muslim religion faith followers; 1093 (61.27%) did not attend school, and 1029 (57.68%) of the respondents had a poor wealth index. The study showed that 1185 (68.10%) of the respondents were still breastfeeding, and 1380 (77.35%) of the respondents reported that they wanted to have two or less ideal numbers of children. Regarding the sex of the household head, 1428 (80.04%) of the respondents were male (Table 1).

**Table 1:** Weighted socio-demographic reproductive and child status-related characteristics of study participants, EDHS, 2019 [*N* = 1784]

Variables	Category	Frequency	Percentages
**Short birth intervals**	No	662	37.11
	Yes	1122	62.89
**Age of the mother**	15–24	339	19.00
	25–34	1051	58.91
	35–49	394	22.09
**Residence**	Urban	354	19.84
	Rural	1430	80.16
**Religion**	Orthodox	390	21.97
	Muslim	1039	58.24
	Protestant	310	17.38
	Others++	43	2.41
**Age at first birth**	Less than 18	843	47.25
	18 and above	941	52.75
**Wealth index**	Poor	1029	57.68
	Middle	244	13.68
	Rich	511	28.64
**Sex of child**	Male	906	50.78
	Female	878	49.22
**Contraceptive utilization**	Use	482	27.02
	No	1302	72.98
**Survival of index child**	No	31	1.74
	Yes	1753	98.26
**Sex of household head**	Male	1428	80.04
	Female	356	19.96
**Respondents education level**	no education	1093	61.27
	Primary	505	28.31
	Secondary	112	6.28
	Higher	74	4.15
**Region**	Tigray	137	7.81
	Afar	245	12.53
	Amhara	111	8.14
	Oromia	232	13.34
	Somali	232	11.31
	Beneshangul Gumuz	152	9.60
	SNNPR	211	11.80
	Gambella	130	7.89
	Harari	140	7.49
	Addis Ababa	66	3.25
	Dire Dawa	128	6.83
Ideal no children	<=2	1380	77.35
	> 2	404	22.65
Breast feeding practice	Never	82	4.71
	Not currently	473	27.18
	Still breast feeding	1185	68.10

### Short birth intervals characteristics among reproductive age women [13–19, 21–25, 27–49]

Out of the total respondents, 1122 (62.89%) (95 CI: 60.62, 65.1%) women have experienced SBI. Women with SBI aged 15–24 years comprised 238 (21.21%), 635 (56.59%) ranged from 25 to 34 years, and 249 (22.19%) were over 35 years.

### Spatial distribution and hot spot (gets-Ord G statistics) analysis of short birth interval

**Fig. 2:**
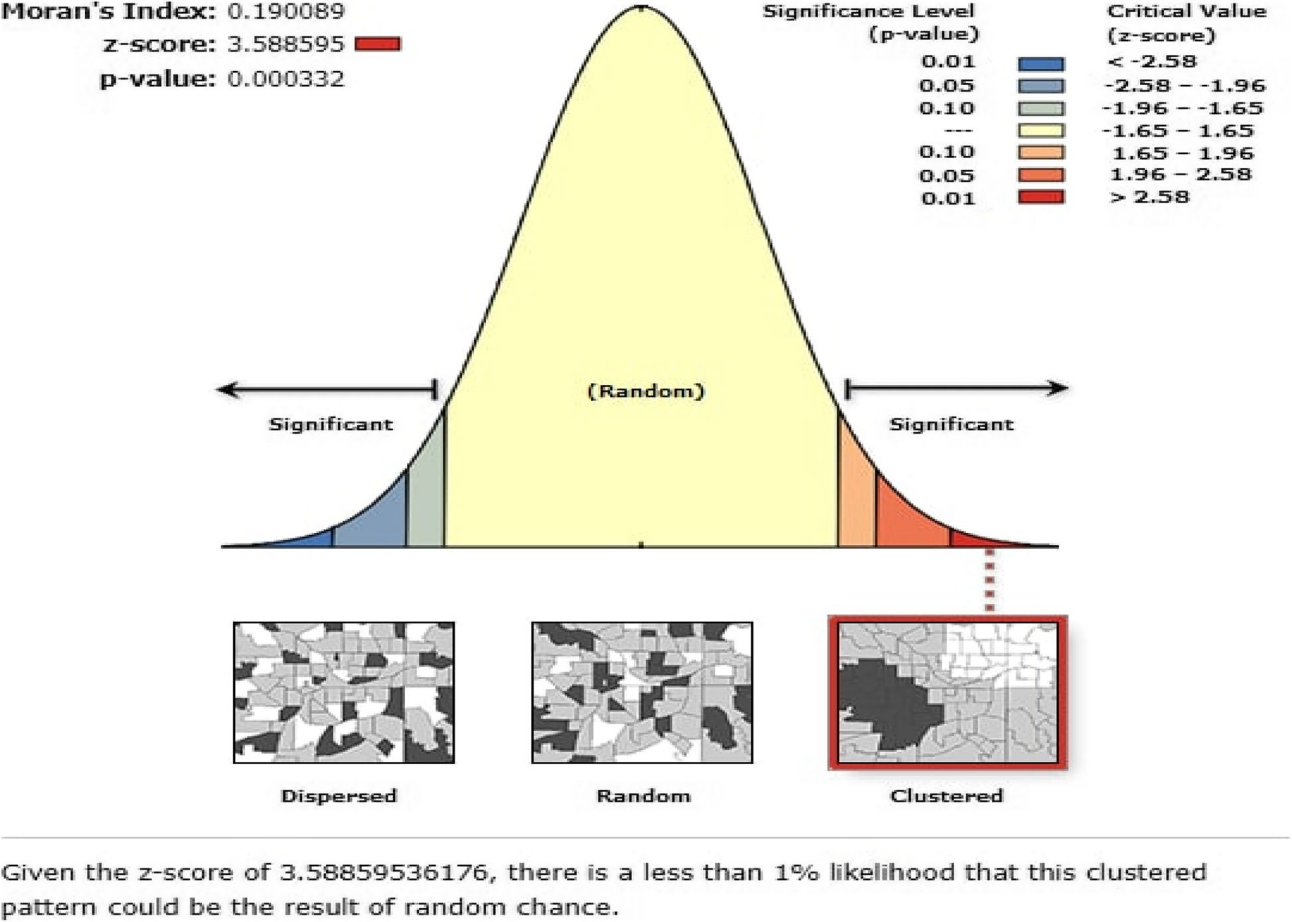
A spatial autocorrelation analysis (Moran’s I) based on feature location and attributed values of short birth interval in Ethiopia based on mini EDHS 2019

The spatial autocorrelation analysis based on the feature locations and attributed values revealed a culturing pattern of short birth intervals (non-random) across a study area (global Moran’s I test was 0.190089 and the Z-score was positive with a *p*-value of 0.0003), indicating that the observed clustering is less than 1% likely to be the result of random chance. The spatial autocorrelation analysis revealed the presence of statistically significant clusters at a 0.01 level of significance (Fig. 2).

**Fig. 3:**
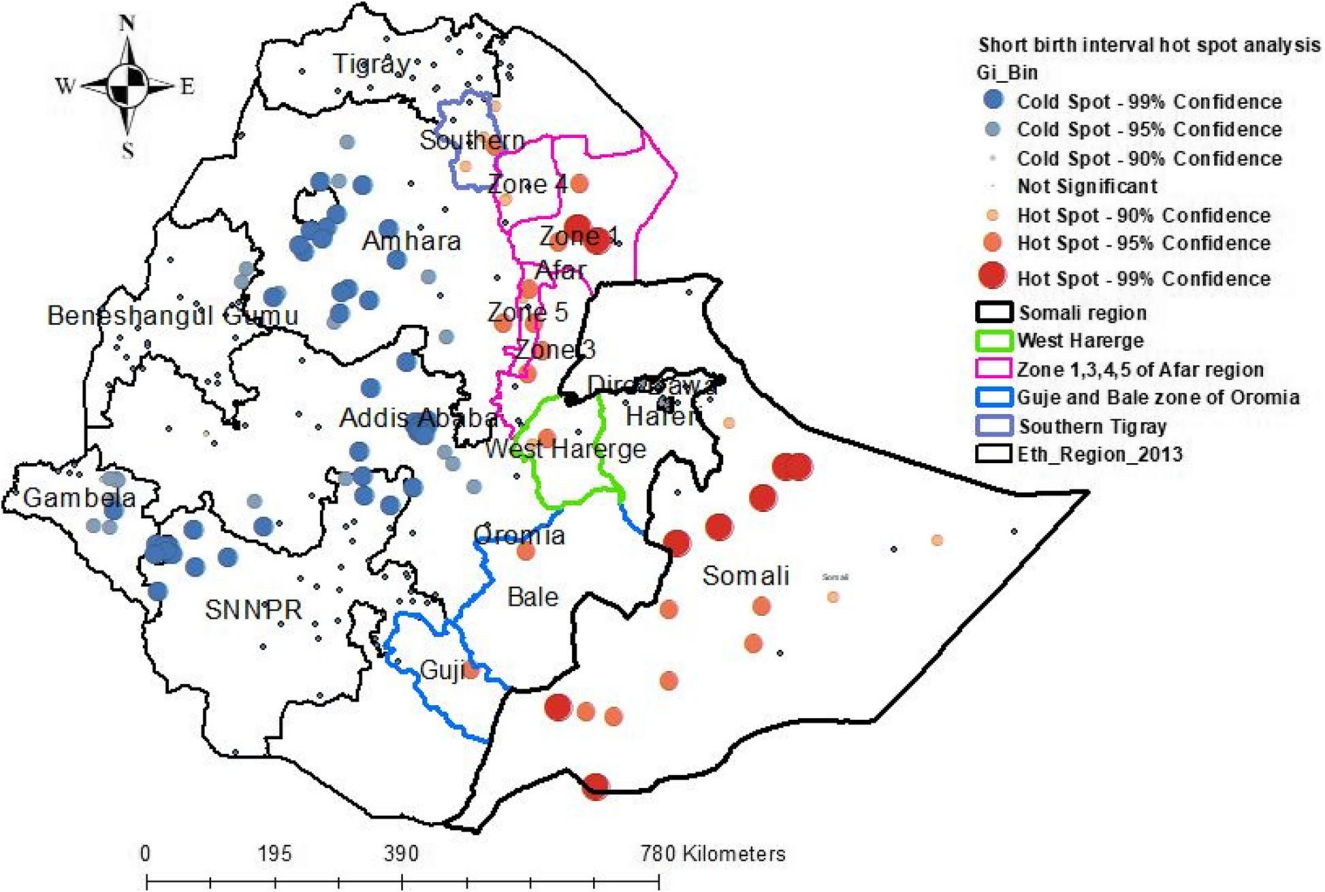
Hot Spot analysis of short birth interval In Ethiopia in EDHS 2019

The Getis–Ord hot spot analysis identifies hot spots (areas with high cases of short birth intervals) and cold spots (areas with low cases of short birth intervals). The hot spots (high risk of a short birth intervals) were significantly clustered in almost all areas of the Somali region, the Oromia region in Bale, Guje, and West Harerge Zones, in the Afar region, specifically in Zone 1, Zone 3, Zone 4, and Zone 5, and southern Tigray. Hence, an evidence-based public health intervention and designing health awareness and promotion should focus on this area to limit the fertility rate of women. Conversely, a cold spot area of the short birth interval was observed in the northern and southwest and central parts of Amhara region, Addis Ababa, the north and northwest of SNNPR, the central and northern parts of Gambella, and some parts of Oromia region (Fig. 3).

### Spatial interpolation

**Fig. 4:**
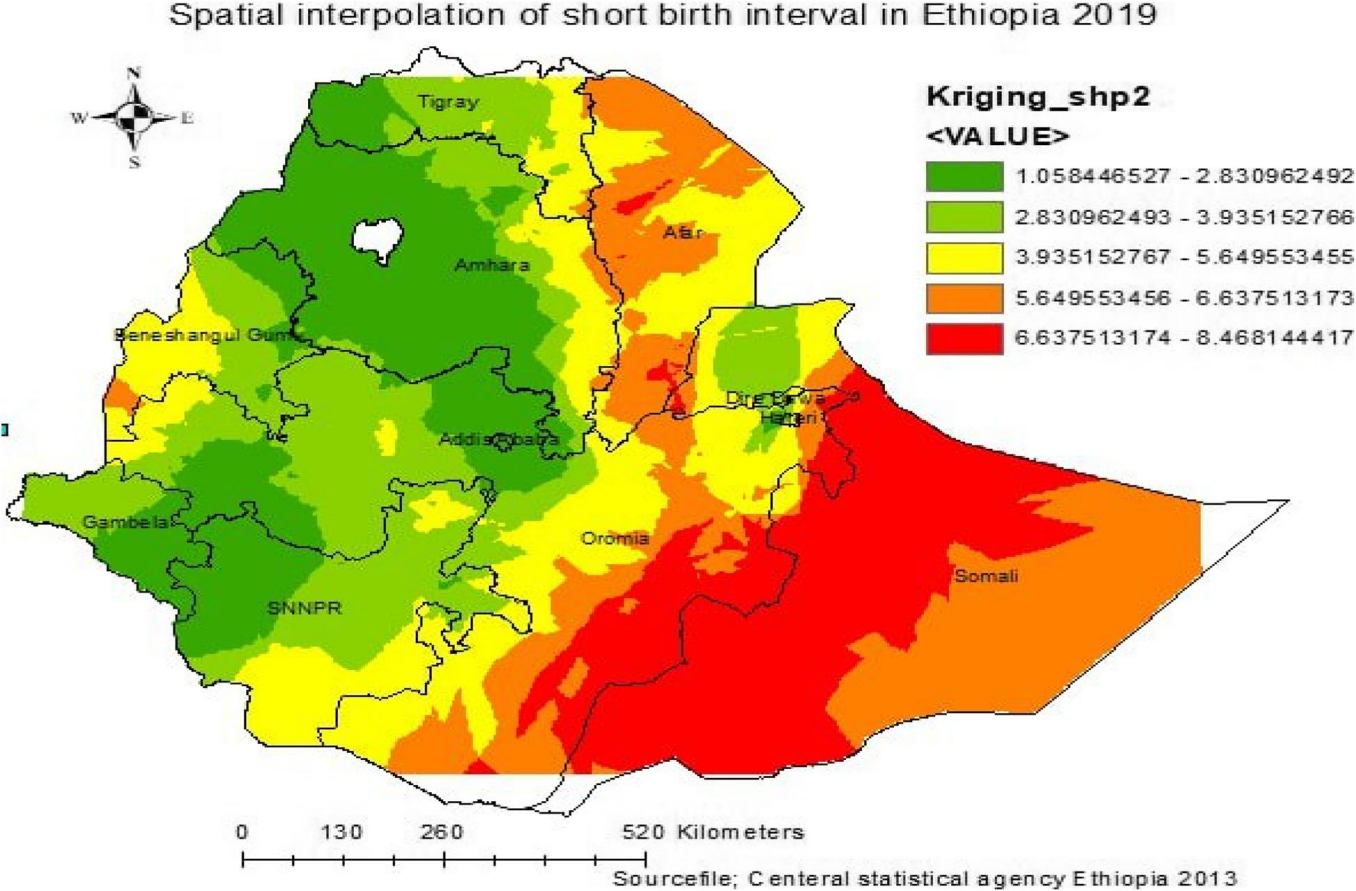
Spatial Interpolation of short birth interval Ethiopia in EDHS in 2019

We estimated the spatial/geographical variation of the short birth interval for areas where data points were not collected across Ethiopia by using the Kriging interpolation method. The produced risk maps highlight the areas with lower and higher risk of short birth intervals. Overall, our prediction map revealed that there is noticeable local geographic variation in terms of short birth intervals, with the risk of short birth intervals being elevated from green to red colored areas. Oversight of the red colored areas that are likely to have higher short birth intervals and mapping the risk of the short birth intervals can help policymakers identify priority areas during community awareness creation and the health education process. In contrast, the predicted areas with a lower prevalence of short birth intervals were detected in Addis Ababa, most parts of Amhara, the south and southeast of Gambella, the northwest of SNNPR, and Dire Dawa (Fig. 4). Predicting areas that are likely to have a short birth interval and mapping the risks of a short birth interval will help FMOH, policymakers, and program managers make evidence-based decisions about public health interventions.

### SaTScan spatial analysis of short birth interval

**Fig. 5:**
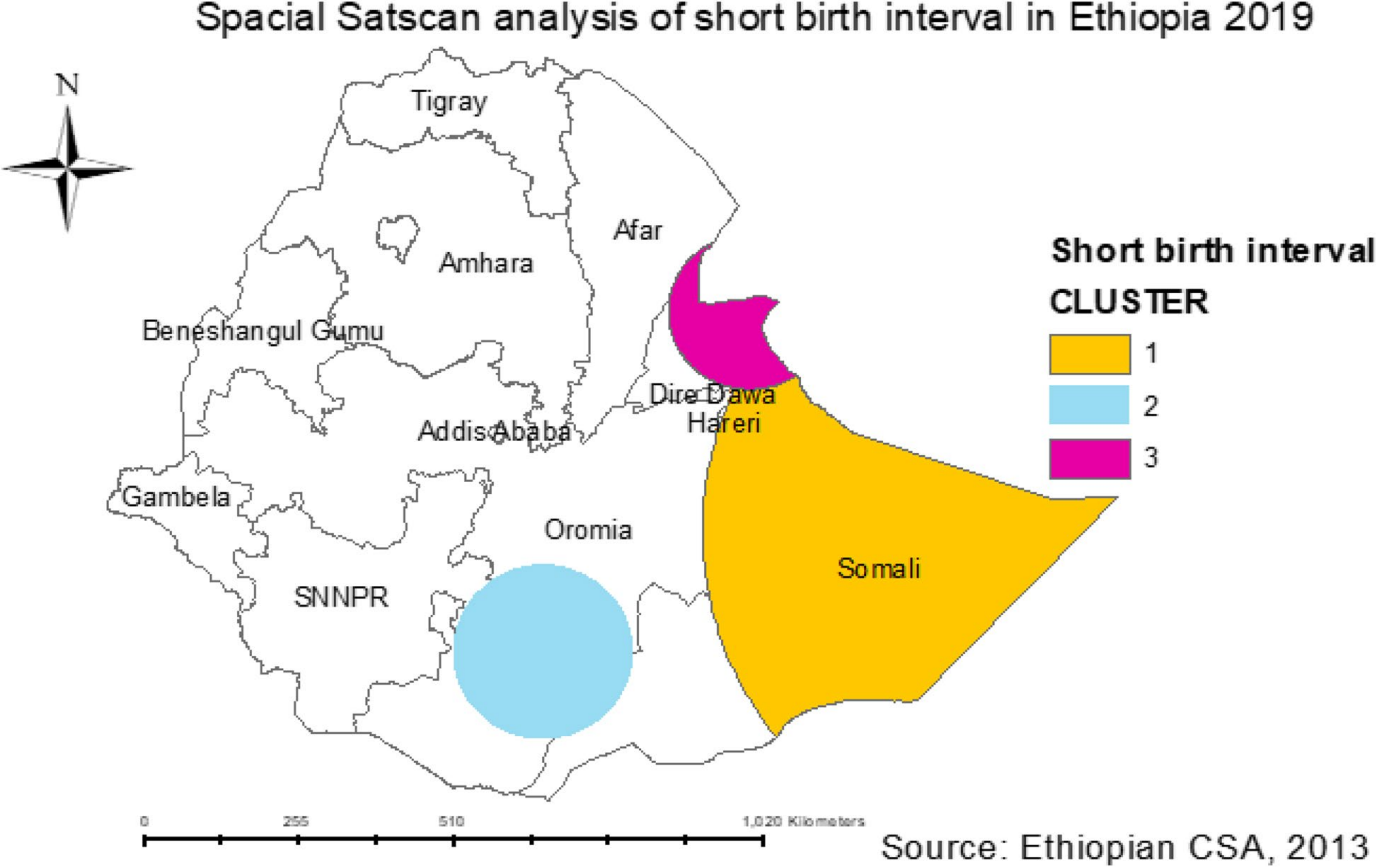
Spatial clustering of areas with short birth intervals in Ethiopia in EDHS 2019

The SaTScan spatial analysis identified one statistically significant SaTScan cluster area. A total sampled population of 494 was found in the primary cluster area using SaTScan analysis with *p*-value value < 0.0000048. This means that the prevalence of short birth intervals was higher inside the SaTScan circular window compared with that outside the SaTScan window. The most likely primary SaTScan cluster of high short birth interval is located in the border area of the Somali region, centered at 6.639662 N, 44.465853 E within a 630.81 km radius. A relative risk (RR) of 1.24 and Log-Likelihood Ratio (LLR) of 18.17373 at a *P*-value < 0.001); (RR = 1.24, LLR = 18.173173, *p* < 0.001). Women whose age was [13–19, 21–25, 27–49] and who lived in the primary cluster were 24% more likely to have a short birth interval than those who lived outside the window (Fig. 5).

### Determinants of short birth interval among reproductive age women in Ethiopia

#### Measure of random effect (model comparison)

According to the null model, the variance of the random factor was 0.22 at a 95% CI (0.04, 0.34), indicating the presence of heterogeneous areas since the variance estimate of short birth intervals is greater than zero. This shows there are enumeration (cluster) difference in short birth intervals among reproductive-age women in Ethiopia. Thus, multilevel analysis should be considered an appropriate approach for identifying both the individual or community-level predictors. The intra-cluster correlation coefficient (ICC), which indicated that 8% of the total variability of short birth intervals is due to differences across cluster areas, with the remaining unexplained 92% attributable to individual differences (Table 2).

**Table 2:** measure of variation (random intercept model) and model fit statistics of short birth interval among reproductive age groups (15–48) woman’s in Ethiopia EDHS 2019

Measure of variation & model comparison	Null model	Model I	Model II	Model III
Variance	0.22	0.03	0.04	0.305
ICC	0.08	0.43	0.35	0.002
LLR	− 1176.6	−10,121.0	−10,763.6	− 9050.3
Model fitness test statistics				
BIC	22,351.019	18,156.78	19,694.84	18,027.08
AIC	21,340.0	18,148	19,623	18,057.77

#### Model fitness statistics

As indicated in Table 2, in the model fitness statistics test, both the values of AIC and BIC reduced in each successive model, and we preferred the AIC values for model comparison. The mixed effect logistic regression model was the best fitted model (Final model).

In the multivariable mixed effect logistic regression analysis model, the educational status of the mother, their age, the sex of the household head, the ideal number of children, religion, and wealth status were found to be statistically significant factors for short birth intervals among reproductive age women in Ethiopia.

After adjusting for other variables, women whose ages were between 25 and 34 and 35–49 were 0.40 (AOR = 0.40, 95% CI: 0.35, 0.45) and 0.44 (AOR = 0.44, 95% CI: 0.38, 0.51) times less likely to have a short birth interval than their counterparts. This study revealed that those women who are Muslim religion followers were 3.5 (AOR = 3.5, 95% CI: 2.7, 4.69) times more likely to have short birth intervals than those who are Orthodox religion followers. Keeping other variables constant, women who have no formal education, who attended primary and secondary education were 0.5 (AOR = 0.5, 95% CI: 0.37, 0.70), 0.4 (AOR = 0.4, 95% CI: 0.28, 0.53), and 0.3 (AOR = 0.3, 95% CI: 0.24, 0.48) less likely to have short birth intervals compared to women who have higher education.

Besides, we have identified that the wealth status of the study participants was significantly associated with short birth intervals. Respondents, women who have middle and rich wealth status, were 1.3 (AOR = 1.3, 95% CI: 1.2, 1.52) and 1.4 (AOR = 1.4, 95% CI: 1.3, 1.68) times more likely to have short birth intervals than their counterparts. The odds of SBI among women who have female sex children was 1.2 (AOR = 1.2, 95% CI: 1.09, 1.42) times more likely to have SBI than those among women who have male sex children. Finally, in this study, women who wanted to have two or fewer children were 0.2 (AOR = 0.2, 95% CI: 0.25, 0.32) times less likely to have short birth intervals than those who wanted more than two ideal numbers of children (Table 3).

**Table 3:** Multivariable multilevel mixed effect logistic regression analysis of factors associated with short birth intervals among reproductive age woman’s in Ethiopia, 2019

Variables	Model 0	Model 1 AOR(95% CI)	Model2 AOR(95%CI)	Model 3 AOR(95%CI)
**Age**				
15–24		1	–	1
25–34		.40(.35, .45)^a^	–	.40(.35, .45) ^a^
35–49		.40(.38, .51) ^a^	–	.44(.38, .51) ^a^
**Religion**				
Orthodox		1	–	1
Muslim		3.4 (2.63, 4.38) ^a^	–	3.5 (2.7, 4.69) ^a^
Protestant		.75(.6, .94)	–	.7 (.59, 1.41)
Others		8.0 (5.66, 14.51)	–	9 (5.6, 14.42)
**Educational level of the mother**				
No education		.51(.28, .53) ^a^	–	.5(.37, .70) ^a^
Primary education		.39(.28, .53) ^a^	–	.4(.28, .53) ^a^
Secondary education		.34(.24, .48) ^a^	–	.3(.24, .48) ^a^
Higher education		1	–	1
**Wealth status**				
Poor		1	–	1
Middle		1.35 (1.2, 1.53 ^a^	–	1.3 (1.20,1.52) ^a^
Rich		1.47 (1.29, 1.67) ^a^	–	1.4 (1.30,1.68) ^a^
**Sex of index child**				
Female		1.42 (1.06, 1.86)	–	1.3 (1.09, 1.42) ^a^
Male		1	–	1
**Sex household head**				
Female		1.24 (1.09, 1.42) ^a^	–	1.2 (0.09, 1.42)
Male		1	–	1
**Survival index child**				
No		1.06(.77, 1.46)	–	1.0(.76, 1.45)
Yes		1	–	1
**Breastfeeding**				
Never		.55(.44, .68) ^a^	–	1.2 (1.01, 2.67)
Still breastfeeding		.37(.33, .41) ^a^	–	.4(.33, 1.41)
Currently breastfeeding		1	–	1
**Ideal no of children**				
<=2		.28(.25, .32) ^a^	–	.2(.25, .32) ^a^
> 2		1	–	
**Contra_ utilization**				
No		1.01(.919, 1.10)	–	1.0(.92,1.11)
Yes		1	–	1
**Region**				
Tigray		–	1	1
Afar		–	1.98(.85, 4.59)	.4(.15, 1.12)
Amhara		–	.40(.18, .86) ^a^	.4(.17, 1.01)
Oromia		–	1.32(.63, 2.79)	.7(.30, 1.75)
Somali		–	3.60 (1.61, 8.03) ^a^	.6(.26, 1.79)
Beneshangul Gumuz		–	1.29(.52, 3.21)	.5(.20, 1.64)
SNNPR		–	.80(.38, 1.68)	.7(.33, 1.89)
Gambella		–	.61(.21, 1.73)	.4(.123,1.39)
Harari		–	1.41(.48, 4.11)	.3(.10, 1.18)
Addis Ababa		–	1.62(.62, 4.24)	.9(.32, 2.92)
Dire Dawa		–	1.42(.53, 3.78)	.3(.12, 1.14)
**Residence**				
Rural		–	1.04(.64, 1.69)	1.3(.74, 2.30)
Urban		–	1	1

^a^ Significant at each model, 1 = Reference category

## Discussion

The purpose of this study was to understand the spatial distribution and predictors of short birth intervals among reproductive-age women of childbearing who have had at least two consecutive births. The overall prevalence of SBI (< 33 months) among women of this age group [13–19, 21–25, 27–49] was 1122 (62.89%). This study was higher than a study reported in Ethiopia, 43.4% [25], 45.7% [26], EDHIS data of selected developing regions of Ethiopia 46% [27], Northern Ethiopia, 23.3% [28], and Lemo district, Southern Ethiopia, 57% [29].

This discrepancy could be due to the inconsistent definition of SBI. Those previous studies considered SBI if the birth interval was less than 24 months, whereas our study defined it as less than 33 months. Besides, this might be due to socio-cultural barriers across the study areas and sample size differences. Moreover, the desire to have more children, which are rooted in the community, has changed due to economic concerns and changes in lifestyle.

In this study, the spatial analysis, spatial autocorrelation, hotspot, and SaTScan analysis were reported to access the spatial distribution of the short birth interval. The spatial autocorrelation analysis in this study confirmed the distribution of short birth intervals was clustered (global Moran’s I test was 0.190089 and the Z-score was positive with a *p*-value of 0.0003). The hotspot analysis (high risk of short birth interval) and (low risk of short birth interval) were observed in some geographical areas. The spatial clustering of hot spots (high risk of a short birth interval) was observed in the most parts of Somali in the Afar region such as: Zone 1, Zone 3, Zone 4, and Zone 5, and Northern Tigray, followed by Oromia region in West Harerge, Guje, and Bale zones. Hence, an evidence-based public health intervention and designing health awareness and promotion should be focus on these areas to limit the fertility rate of women. A cold spot area of the short birth interval was observed in the northern and southwest and central parts of Amhara region, Addis Ababa, the northern and northwest parts of SNNPR, the central and northern parts of Gambella, and some parts of Oromia region.

The SaTScan spatial scan statistical analysis identified the most likely primary significant cluster of short birth intervals. The most likely primary SaTScan cluster of high short birth interval areas was detected in Somali and revealed that women aged women [13–19, 21–25, 27–49] and who lived in the primary cluster were 24% more likely to have a short birth intervals than those who lived outside the window. This might be due to low utilization of modern contraceptives and less government intervention through health education and creating awareness about birth spacing.

In multilevel analysis, the age of the mother, religion, education, wealth status, sex of the indexed child, and the ideal number of children were found to be significant factors in short birth intervals. Participants who follow the have more SBI when compared to Orthodox Christians. This study’s finding is supported by a study done in Ethiopia, Bangladesh, and Nigeria [1, 52]. This might be due to the low utilization of contraceptive methods among Muslim religious followers.

A study done in Bahir Dar, Ethiopia, reported 63.9% of Orthodox Christian followers versus 36.1% of Muslim followers using modern contraceptives [53]. The FMOH, program managers, and policy developers need to focus on creating awareness regarding the utilization of contraceptives among Muslim religious followers. This study revealed that women having female sex of the index child had a higher risk of short birth intervals than those who had male sex of the index child. This study is supported by the previous study reported in Ethiopia [33, 38]. This could be due to the patent and community’s preference to have male children rather than to have female children. The government and FMOH should work to change this community traditional believes about child preferences since it contributes to population growth. As a result, public awareness creation and health education need to be strengthened.

The maternal age was found to be a significant predictor of short birth intervals, with younger women having shorter birth intervals than older women. This finding is supported by a study done in Ethiopia. Younger women have a high rate of fertility as compared to older women. Another reason might be younger women wants to build their desired family size, which could probably decrease their birth spacing.

Surprisingly, this study found that women with no formal education, primary, and secondary education had a low risk of short birth intervals compared to women having higher educational status. Most studies reported women who attained higher education had longer birth intervals as compared to women with low education or no formal education. This could be the hypothesis: that women who attained higher education get married later in life and subsequently wants to make a family. Additionally, this might be due to the fact that educated women may wish to compress or limit childbearing into fewer years and focus on educational activities. However, further qualitative and quantitative study may be required to investigate the evidence, especially in developing countries.

The current study suggests that women who wanted to have fewer children had a lower risk of short birth intervals than those who wanted more. This could be due to, women may have achieved their desired family size and may feel less pressure or be in less of a hurry to get pregnant again. The wealth status of the women was found to be a predictor of birth interval spacing. Respondents, who have middle and rich wealth status, were 30 and 40% more likely to have short birth intervals than women with poor wealth status. Which is inconsistent with a study reported in Ethiopia [34, 35]. According to the EDHS 2005, it suggests that the median of moths increased with increased wealth status [54]. However, the discrepancy in this finding might be that women with poor wealth status spend their time on their jobs to fulfil their basic needs.

## Conclusion

The prevalence of short birth intervals in Ethiopia is still optimally high. This study also observed a clustered pattern of short birth intervals in Ethiopia. Statistically significant local primary clusters of high SBI areas were detected in the Somali region. The individual-level characteristics of women’s educational level, age, religion, wealth status, the sex of the indexed child, and the ideal number of children were statistically significant factors of SBI. Therefore, the federal ministry of health and other concerned maternal and child health program managers give a concern about the areas with high SBI coverage identified in this study. It is also better to consider the individual determinant factors that may help planners, policymakers, and decision-makers with an evidence-based health intervention.

## Strength and limitation of the study

This study used a multilevel mixed effect model and EDHS national datasets to examine the effects of clustering, which is considered the strength of this study. This study shares recall bias since the data were collected retrospectively and the 2019 EDHS data sets were not observed since some important variables that determine short birth intervals were not included in this study.

## Data Availability

The dataset for this study is available on the Measure DHS program and you can access the dataset freely it from (http://dhsprogram.com) website, after clearly explaining the objective of the study, the data generated and analyzed for this study are included in the form of tables, maps, and texts.

## Abbreviations

AIC: Akaike’s Information Criteria
AOR: Adjusted Odds Ratio
BIC: Bayesian Information Criteria
CI: Confidence Interval
CSA: Central Statistical Agency
COR: Crude Odds Ratio
DHS: Demographic and Health Survey
EAs: Enumeration Areas
EDHS: Ethiopian Demographic and Health Survey
EMDHS: Ethiopia Mini Demography and Health Survey
EPHC: Ethiopian Population and Housing Census
EPHI: Ethiopian Public Health Institute
ICC: Intra-class Correlation Coefficient
ICF: International Classification of Functioning, Disability and Health
RR: Relative Risk
LLR: Log likelihood Ratio
SBI: Short birth interval
SNNPR: South Nations Nationalities and People’s Region
STATA: Statistical Software for Data Science
USAID: United States Agency for International Development
WHO: World Health Organization
